# “Just one word, plastic!”: Controversies and caveats in innate lymphoid cell plasticity

**DOI:** 10.3389/fimmu.2022.946905

**Published:** 2022-08-16

**Authors:** Ahmed Kabil, Samuel B. Shin, Michael R. Hughes, Kelly M. McNagny

**Affiliations:** ^1^ School of Biomedical Engineering, University of British Columbia, Vancouver, BC, Canada; ^2^ Department of Medical Genetics, University of British Columbia, Vancouver, BC, Canada; ^3^ Centre for Heart and Lung Innovation (HLI), St Paul’s Hospital, University of British Columbia, Vancouver, BC, Canada

**Keywords:** plasticity, migration, mucosal immunology, inflammation, innate lymphoid cell (ILC), controversy, transdifferentiation, local progenitors

## Abstract

Innate lymphoid cells (ILCs) are frontline immune effectors involved in the early stages of host defense and maintenance of tissue homeostasis, particularly at mucosal surfaces such as the intestine, lung, and skin. Canonical ILCs are described as tissue-resident cells that populate peripheral tissues early in life and respond appropriately based on environmental exposure and their anatomical niche and tissue microenvironment. Intriguingly, there are accumulating reports of ILC “plasticity” that note the existence of non-canonical ILCs that exhibit distinct patterns of master transcription factor expression and cytokine production profiles in response to tissue inflammation. Yet this concept of ILC-plasticity is controversial due to several confounding caveats that include, among others, the independent large-scale recruitment of new ILC subsets from distal sites and the local, in situ, differentiation of uncommitted resident precursors. Nevertheless, the ability of ILCs to acquire unique characteristics and adapt to local environmental cues is an attractive paradigm because it would enable the rapid adaptation of innate responses to a wider array of pathogens even in the absence of pre-existing ‘prototypical’ ILC responder subsets. Despite the impressive recent progress in understanding ILC biology, the true contribution of ILC plasticity to tissue homeostasis and disease and how it is regulated remains obscure. Here, we detail current methodologies used to study ILC plasticity in mice and review the mechanisms that drive and regulate functional ILC plasticity in response to polarizing signals in their microenvironment and different cytokine milieus. Finally, we discuss the physiological relevance of ILC plasticity and its implications for potential therapeutics and treatments.

## Introduction

The innate lymphoid cell (ILC) family is a heterogeneous group of recently discovered immune-modulatory cells at the centre of extensive research. ILCs are now widely accepted to fulfill a similar set of biological functions to their more extensively studied relatives, CD4^+^ helper (Th) and CD8^+^ cytotoxic T cells (T_CTL_). Yet, they perform these complementary roles in the absence of an antigen-specific receptor [i.e., functional T cell receptor (TCR)] and thus, represent an innate branch of this lymphocyte family (1). Besides the well-known and well-characterized cytotoxic innate lymphoid cells, namely natural killer (NK) cells, the more recently discovered groups of cytokine-producing “helper-like” ILC lineages were identified by several laboratories between 2008-2010 (2–16).

NK cells primarily function as cytotoxic cells that circulate in the bloodstream and can be thought of as an innate branch of lymphocytes serving parallel functions to CD8^+^ T_CTL_ (17). “Helper-like” ILCs (ILC1, ILC2, and ILC3) are mainly tissue-resident with the capacity to migrate in response to inflammation (18, 19). Each ILC subset has distinct functional capabilities (20); ILC1s are activated in response to interleukin (IL)-12, IL-15, and IL-18 and primarily produce interferon gamma (IFNγ), which is associated with defence against viruses and intracellular bacteria. ILC2s are activated by IL-25, IL-33, or TSLP and, in response, produce type 2 cytokines, mainly IL-5 and IL-13, which are important in promoting allergic reactions but also serve as barrier surface defence mechanisms to eliminate parasitic infections. ILC3s are activated in response to IL-1β or IL-23 and produce IL-17 and IL-22, which are important for defence against extracellular pathogens, including bacteria and fungi. The principal activators and effector cytokines of different ILC subsets are of interest because accumulating evidence suggest that they are critical for tissue repair and homeostasis, metabolic regulation, and in neuroimmune circuits with enteric neurons (1). Despite the role of ILCs within the multi-layered regulation of barrier defenses, they have also been associated with tissue pathology in several inflammatory diseases, including allergic asthma, dermatitis, psoriasis, and intestinal inflammation (21).

**Figure 1:**
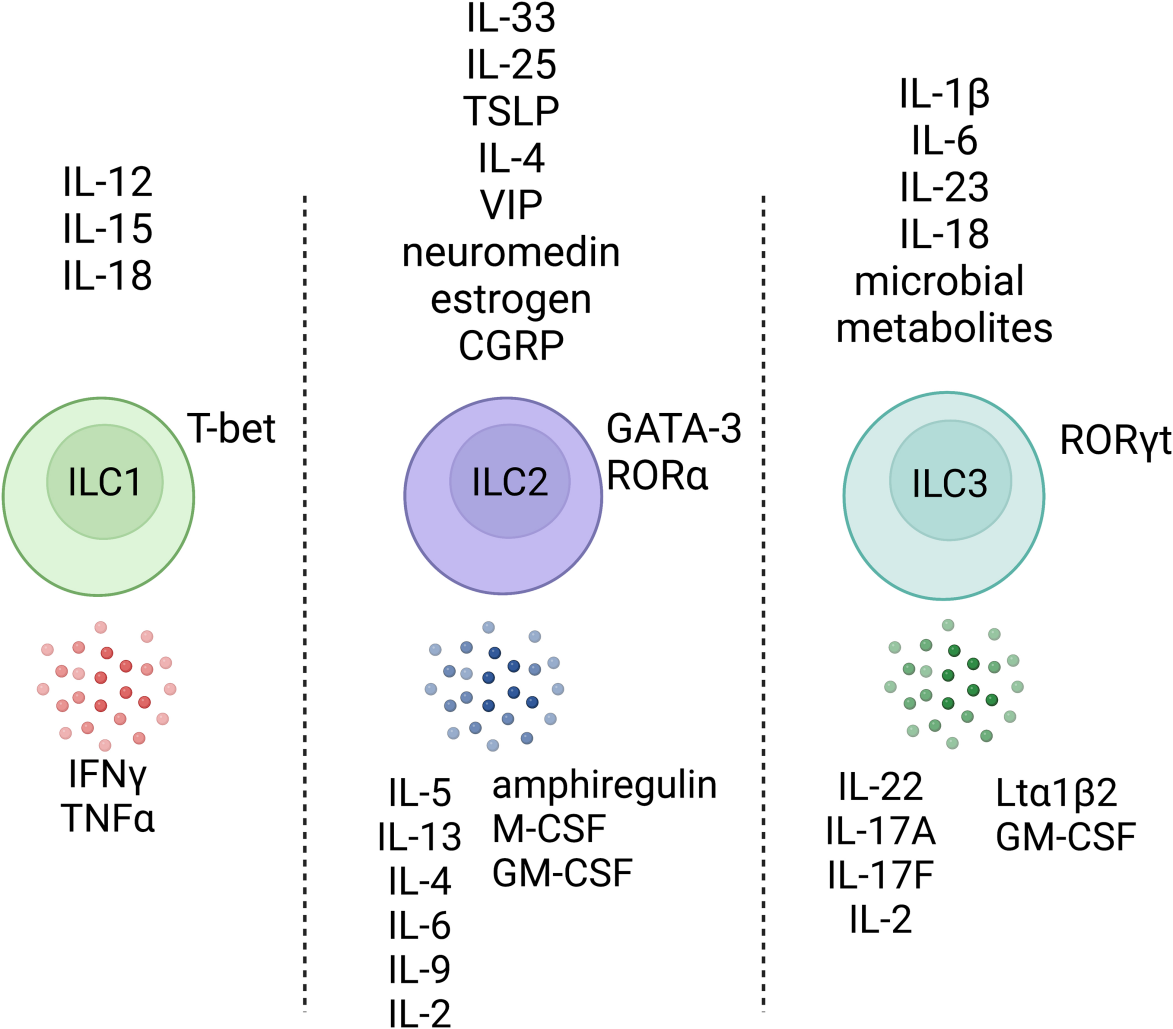
Helper-like innate lymphoid cells respond to various environmental factors. ILCs do not express an antigen-specific receptor but can respond to various environmental factors and, upon stimulation, produce effector cytokines that pattern the stereotype modules that the immune system has evolved to interact with tissues, the outside world, and organisms. Created with Biorender.org.

ILCs populate the peripheral tissues very early in ontogeny (e.g., embryonic day (E) 12.5 – E13.5 in mice) and are postnatally activated at the time of birth, serving as an early guard against infection (22–24). ILCs are most frequently observed in tissues, particularly at barrier surfaces of tissues including the gut, skin, and lungs, and respond to various rapidly released damage-associated microenvironmental factors (collectively termed “alarmins”), neuropeptides, and cytokines (**Figure 1**) (25). Acting as danger signals, alarmins are typically secreted by local tissue epithelial cells and stroma in response to local epithelia insults or infection. As first responders, tissue-resident ILCs then interpret these immediate signals and orchestrate the subsequent, appropriate downstream immune effector cell response (26, 27). ILCs are poised to provide a rapid response because they acquire chromatin accessibility for critical effector cytokine genes in the early stages of cellular maturation and development, prior to activation (28). By contrast, naïve T cells need to be primed, activated, and appropriately polarized in lymph nodes prior to the subsequent migration to effector sites, making their response relatively slow. Therefore, ILCs represent a local source of appropriate effector cytokines during the earliest stages of infection, providing a first innate response that allows critical time for the more targeted T cells to become instructed and recruited (29).

Mature ILCs are typically defined by T cell and NK cell lineage-determining transcription factors, and the lack of lineage-specific surface markers such as CD14 (myeloid cells), CD49b (NK cells), CD19 (B cells), CD3 (T-cells), among others (30). ILC1s are dependent on the canonical Th1-type transcriptional regulator, T-bet (*Tbx21*), but not the conventional NK (cNK) transcription factor EOMES (31, 32). ILC2s are characterized by the high expression of GATA3 and RAR-related orphan receptor alpha (RORα) (33–35). ILC3s express the Th17-associated transcription factor RAR-related orphan receptor gamma (RORγt). Although these properties initially guided the classification of ILCs into subsets and their placement within the hematopoietic phylogenetic tree, an influx of single cell studies has begun to reveal a complex network between different ILC subgroups as well as possible evidence of plasticity between specific subclasses of ILCs under certain conditions (36–40). Notably, these studies demonstrate that ILCs are likely much more heterogeneous than initially thought, even within subgroups that express the same “master” transcription factors, possibly reflecting more fine-tuned gene expression linked to their tissue microenvironment and rapid changes in response to the local inflammatory milieu (**Table 1**).

**Table 1:** Summary of cell surface phenotype, transcription factor/gene expression profiles of mouse ILC2 and ILC3 subsets.

ILC2 and ILC3 subsets	Transcription factor and gene expression profile*	References
Conventional ST2^+^IL-18R^-^ ILC2 (nILC2)	*Gata3* ^high^ *Rora* ^+^	(34, 41, 42)
Resident ST2^-^ IL-18R^+^ ILC2 progenitors	*Gata3* ^int^ *Tcf7* ^high^ *Rora* ^+^	(43, 44)
Ex-ILC2/ILC1-like cells	*Gata3* ^int^ *Tbx21* ^+^	(45, 46)
Mixed ILC2-ILC3 like cell	*Il5^+^Il13^+^Il17a^+^ Il22^+^Il23r^+^*	(47)
Inflammatory ILC2 (iILC2)	*Gata3* ^high^RORγt^int^ *Il17rb* ^high^	(48–51)
Memory ILC2	*Gata3* ^high^ *Il17rb* ^+^	(52)
CCR6^-^NKp46^-^ DN (precursor-like) ILC3	RORγt^+^ T-bet^-^	(53–56)
Transitional DN ILC3	RORγt^+^T-bet^+^	(53, 55)
NCR^+^ ILC3	RORγt^+^T-bet^+^	(10, 57)
CCR6^+^ LTi-like ILC3	RORγt^+^T-bet^-^	(58, 59)
Ex-ILC3/ILC1	RORγt^-^T-bet^+^	(32, 53, 54, 60)

int – intermediate expression level.

*evidence for transcript expression in italics.

Increasing evidence suggests that ILC subsets are not intrinsically stable and can exhibit considerable plasticity *in vivo* and *in vitro*, most notably with the transdifferentiation of ILC3s into ILC1s through the upregulation of the canonical type 1 transcription factor T-bet and downregulation of RORγt (32, 53, 54, 61, 62). Nevertheless, other studies have reported that during inflammation, ILCs can acquire the ability to egress from the tissues of steady-state residence, enter the circulation, and travel to different anatomical locations and this can serve as a confounder in many studies examining apparent plasticity (48, 49, 63). Additionally, similar ILC subsets from distal tissues have distinctive patterns of tissue-specific gene signatures; for example, IL-33 receptor expression is largely limited to ILC2s in the lungs, whereas the IL-25 receptor is highly expressed by ILC2s in the small intestine and not by lung ILC2s (37). Therefore, the apparent upregulation of novel markers by resident cells, may, in fact, simply reflect the orchestrated recruitment of peripheral cells with a distinctive phenotype (48, 50). Thus, true evidence of plasticity is critically dependent on the use of a combination of epigenetic and transcriptional analyses at the single-cell level coupled with methods for lineage tracing. These key mechanistic details are critical to understanding ILC function in health and disease and their manipulation in designing new avenues for therapy.

In the current review, we focus on the phenomenon of ILC plasticity in mouse models which, through extrapolation to humans, could have significant clinical applications, specifically in the treatment of disease. Moreover, we discuss the heterogeneity and migratory responses of ILCs, and how these properties can impact immunity. As potent immune modulators, ILCs are a double-edged sword and, accordingly, understanding the mechanisms that regulate ILC maturation, recruitment to distal tissues, and plasticity is vital to progress in this field.

## ILC2 plasticity in the lungs: A different perspective

**Figure 2:**
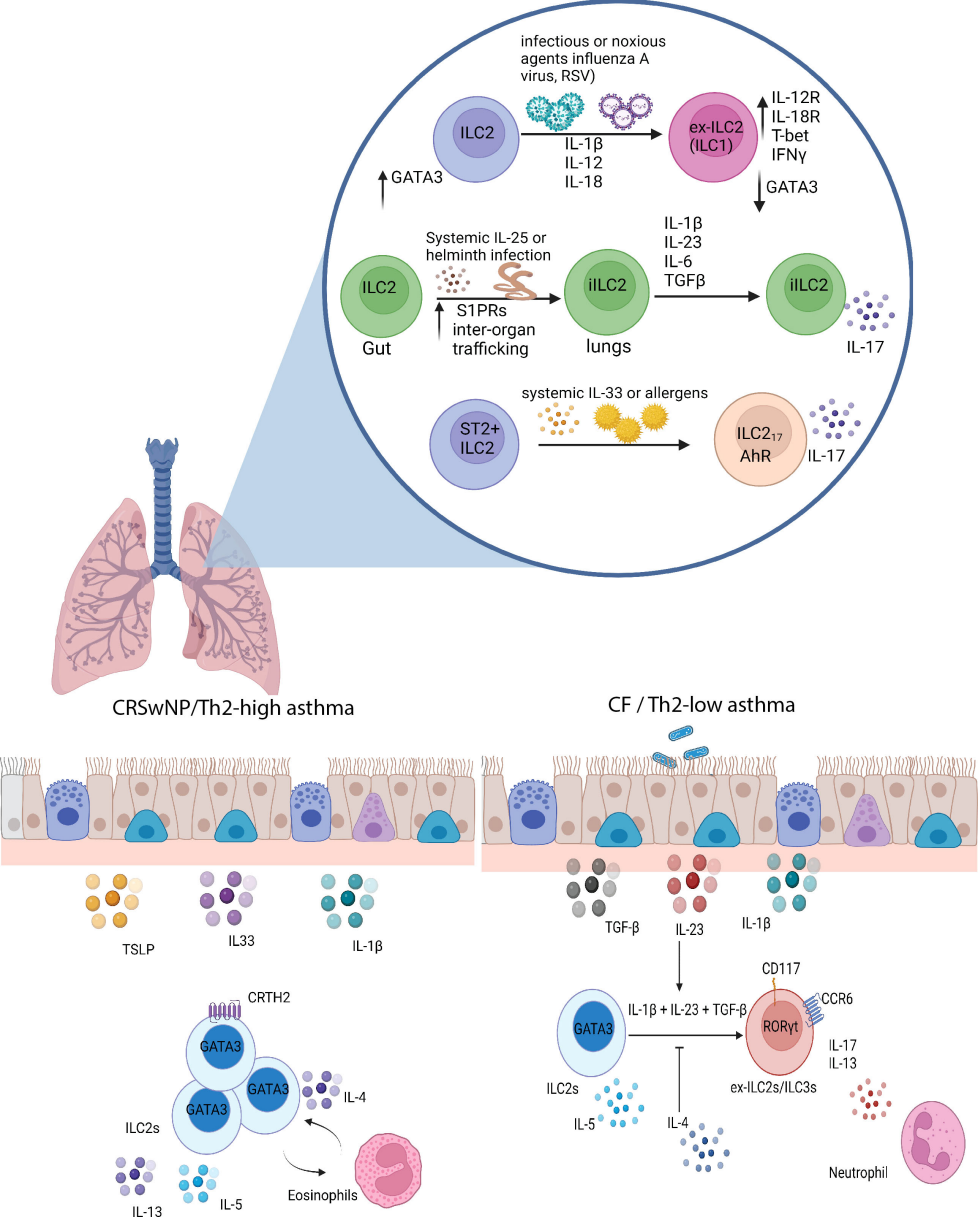
ILC2 plasticity in the lungs. In diseases like COPD and upon viral infections, ILC2s, in response to a combination of IL-12 and IL-1β, can transdifferentiate into IFNγ-producing ILC1-like cells. Infection with the migratory helminth, *Nippostrongylus brasiliensis*, or intraperitoneal IL-25 administration elicits the proliferation of IL-25-responsive intestinal iILC2s, which upregulate S1P receptors and can migrate to systemic sites such as the lungs. These iILC2s have the ability to produce IL-17 in response to Th17-like culture conditions, consisting of TGF-β, IL-1β, IL-23, and IL-6. Other mechanisms have been described by which ILC2s can produce IL-17 in a RORγt-independent manner under the influence of systemic IL-33 or allergen administration. In Th2-high asthma and CRSwNP, ILC2s are conventional players involved in IL-5 and IL-13 production and can interact with eosinophils. However, in cystic fibrosis and possibly Th2 low asthma, ILC2s have the ability, in response to IL-1β, IL-23, and TGF-β, to differentiate into ILC3-like cells that no longer produce IL-5 but retain the capacity to produce IL-13 and IL-17, and in this manner contributes to the neutrophilic inflammation observed in these types of diseases. Created with Biorender.org.

ILC2s are the most studied ILC subset of the airways, likely because the healthy specific-pathogen free (SPF) mouse lung is almost entirely dominated by GATA3^high^ ILC2s and very few RORγt-expressing ILC3s (42, 64). The first functional assessments of lung-resident ILC2s through *ex vivo* pharmacological restimulation assays revealed them to be potent Th2 cytokine producing cells that failed to produce IFNγ or IL-17A (41). These C57BL/6 (B6) or B6-*Rag1* ^-/-^ lung-resident ILC2s (Lineage^-^CD127^+^CD90^+^ cells) expressed the IL-33 receptor (IL-33R/T1-ST2) and were responsive to IL-33, a particularly potent alarmin that, *in vivo*, induces accumulation of ILC2s in the lung and the production of type 2 signature cytokines such as IL-13 and IL-5 (41, 42, 65). More recently, however, in the context of airway diseases and under certain context-dependent perturbations, ILC2s have been observed to undergo an apparent fate shift towards IFNγ-producing ex-ILC2/ILC1s or towards IL17-producing ILC3-like cells (**Figure 2**) (45, 46, 50, 66).

In a mouse model of influenza infection (A/FM/1/47: H_1_N_1_-adapted mouse strain), Silver and colleagues observed a striking increase in the proportion of lung T-bet^+^ ILC1s and a corresponding decrease in the frequency of GATA3^+^ ILC2s (46). To investigate whether the resident ILC2 pool converts into ILC1s, lung ILC2s (ST2^+^IL-18Rα^−^) were sorted from ST2-GFP reporter mice and adoptively transferred into lymphocyte-deficient *Rag2* ^−/−^ *Il2rγ* ^−/−^ mice. Upon influenza infection of the recipients, GFP^+^ ILC2s were found to have downregulated GATA3 expression and upregulated IL-12Rβ2 and IL-18Rα, becoming so-called ex-ILC2s that produced IFNγ upon *ex vivo* stimulation with IL-12 and IL-18. ST2^-^IL-18Rα^+^ ILC1s isolated from mice treated with IL-12, IL-18 and IL-33 had higher expression of *Tbx21*, *Ifng*, *Il12rb2*, and *Il18r1* and lower expression of ILC2-associated transcripts, including *Gata3, Il4, Il5*, and *Rora.*

In line with these findings, Ohne et al. demonstrated that mice treated with intranasal IL-12 or a combination of IL-1β and IL-12 induced a novel T-bet^+^ IL-18Rα^+^ subpopulation within the GATA3^+^ ILC2 population (45). Their data suggest that, in the presence of IL-1β, IL-12 induces an ILC2 functional and phenotypic switch into IFNγ-producing ILC1-like cells that retain the ability to produce IL-13. Despite the co-induction of T-bet and IFNγ expression after *in vitro* stimulation with IL-12 and IL-18, the authors determined that T-bet was dispensable for this switch since similar phenotypic changes were observed in T-bet deficient mice during viral challenge (ie, IL-12Rβ and IL-18Rα upregulation and downregulation of GATA3) (46). T-bet did, however, prove essential for maximal production of IFNγ. Although reduced GATA3 expression was enough to drive ILC2 plasticity, this one-factor model may oversimplify ILC2 diversity given the different phenotypes and tissue-specific gene signatures in different tissues (37).

It is essential to bear in mind that apparent phenotypic and functional “plasticity” could, in fact, be due to the large-scale recruitment of new ILC subsets from distal sites or the differentiation of uncommitted, tissue-resident ILC precursors (67, 68). Indeed, recent work using *Rora*-YFP lineage tracer mice and scRNA-seq of all lung CD45^lo/+^Lineage^-^ cells demonstrated that ILC2s, expressing *Id2, Gata3, Rora, Il7r, Thy1* and lacking *Rorc* and *Tbx21*, can be segregated into two subsets: conventional IL-18Rα^−^ST2^+^ ILC2s and a small subset of IL18Rα^+^ST2^−^ cells that do not produce IL-5 and IL-13 in response to papain- or IL-18-induced mice (43). This latter IL18Rα^+^ST2^−^ ILC subset expresses *Tcf7*, like BM ILC progenitors (ILCps) (69, 70), and can give rise to multiple ILC lineages *in vivo* and *in vitro*. Therefore, instead of plasticity, it is likely that this local progenitor-like population undergoes tissue-specific adaptations *in situ* and differentiates into different effector ILC subsets, including ILC1s or ILC3s, depending on the class of alarmins they are exposed to and the downstream cell-to-cell interactions or signal transductions they detect upon inflammatory challenge.

Several other unanswered questions and caveats remain in the field of ILC2 plasticity in the lungs. Given that *in vitro* stimulation of gut ILC2s with IL-12 and IL-18 does not induce the upregulation of T-bet, there is the question of whether phenotypic changes in the lung represent a tissue-specific phenomenon (71). Indeed, different organs and tissues may be more permissive to induced plasticity or altered transcriptional profiles of a given ILC subset. In addition, different mouse strains with distinct genetic backgrounds may also underlie some variability observed in divergent phenotypes owing to their inherent genetic proclivity for Th1- or Th2- immunity. For example, the mouse-adapted influenza model PR8 induces a greater number of CD90^+^CD25^+^ ILC2s, which are essential in the amphiregulin-dependent reparative process following exposure to this cytopathic virus (41). This finding contradicts the phenotypic switch observed by Silver et al, in which lung resident ILC2s downregulate GATA3 and convert into IFNγ-producing ILC1-like populations in response to infection with PR8 influenza virus (46). Additionally, it is important to note that, in the study by Silver et al., ST2^+/GFP^ mice on a BALB/c background, known to be genetically Th2-skewed, were used to survey the plastic behaviour of ILC2s instead of C57BL/6 mice which have a more balanced Th1/Th2 immune response (72). Thus, it remains unclear to what extent this phenotypic plasticity is a host strain-dependent phenomenon.

It is important to note that ILC2s are functionally heterogeneous. The notion of ILC2 functional plasticity was first demonstrated through intraperitoneal administration of IL-25 or infection with the migratory helminth *Nippostrongylus brasiliensis*. Both treatments in mice induce the expansion of an ST2^-^KLRG1^high^ ILC2 population in the lung termed “inflammatory ILC2s” (iILC2s) (50). This study revealed that these cells are absent during homeostasis in the lungs and are insensitive to both endogenous and exogenous IL-33. Instead, ST2^-^KLRG1^high^ iILC2s express IL-25R (*Il17rb*), low levels of CD90 (Thy-1) compared to ST2^+^ lung-resident “natural” ILC2s (nILC2s) and intermediate amounts of RORγt accompanied by a robust expression of GATA3. The dual expression of RORγt and GATA3 hinted that these iILC2s may function both as type 2 and type 3 cytokine producing cells (50, 73). Predictably, under Th17-like culture conditions in the presence of TGF-β, IL-1β, IL-23 and IL-6, these iILC2s acquired the ability to produce IL-17 while maintaining the ability to produce IL-13, suggesting that lung iILC2s do indeed have the flexibility to become ILC3-like cells (**Figure 2**) (50).

To investigate the possibility that nILC2s can transition into IL-17-expressing iILC2s, nILC2s were exposed to various activating signals *in vitro* including the Notch activator, delta-like ligand (Dll1). Zhang et al. showed that the presence of the Notch ligand, Dll1, induces sorted nILC2s to downregulate *Il1rl1* and upregulate *Il17rb* and convert into IL-13 and IL-17 producing iILC2s. Without adequate Notch signaling, this transition is abolished (73). Although these studies further support the concept of ILC2 plasticity, the true extent of nILC2 to iILC2 conversion *in vivo* can only be addressed definitively by barcoding and fate-mapping approaches of lung resident nILC2s during conversion. Interestingly, IL-33-activated, or allergen-experienced nILC2s can upregulate *Il17rb* (IL-25R) upon re-exposure to papain or IL-33 and become potent IL-5^+^IL-13^+^ “memory” ILC2s, which are capable of responding more quickly and robustly during secondary exposures or after treatment with intranasal IL-25, unlike naïve nILC2s not previously exposed to IL-33 (52). It is less clear whether memory ILC2s can transition into cells that produce both type 2 cytokines and IL-17, especially in IL-17-dependent airway inflammatory models such as obesity-related airway hyperreactivity or oral *Candida albicans* infection model (50, 74).

In addition to IL-17 producing iILC2s, IL-17 producing ST2^+^ ILC2s (ILC2_17_s) have been documented as the main source of IL-17 in the context of papain challenge or IL-33 induced lung inflammation (75). Systemic administration of IL-33 into C57BL/6-wild-type or *Rag1^-/-^* mice resulted in the accumulation of IL-17-producing, Lineage^-^ (CD3, B220, CD5, Gr-1), GATA3^+^ ILC2s in the inflamed lung, which retain the ability to express IL-5 and IL-13. While these ST2^+^ ILC2_17_s are reminiscent of iILC2s in terms of their cytokine profile (50), the former was dependent on the aryl hydrocarbon receptor (AhR) and not RORγt for IL-17 production as they were not responsive in *Ahr*-deficient mice (75). Despite *Rag1^-/-^Rorc^gfp/gfp^* mice exhibiting a lack of ILC3s (another source of IL-17) and having similar pathogenic outcomes in IL-33 induced lung inflammation (75), IL-17-producing ILC3s can still play an important role in different disease contexts such as obesity-induced asthma in which they can mediate the development of airway hyperreactivity and thus, should be at the forefront when considering IL-17-producing ILC populations (74). Moreover, the epigenetic circuits that control the conversion of resting ILC2s into IL-17 producing cells are not well understood and whether lung resident ILC2s are indeed poised to become IL-17 producing cells remains unknown. Certainly, future studies are needed to further clarify the relative contributions of the pool of IL-17-producing ILCs in response to tissue perturbation.

There are several possible approaches to address the extent of ILC2 plasticity in the lungs more adequately. Although ILCs do not express functional T cell receptors, we have previously shown that, surprisingly, lung ILC2s exhibit similar TCR rearrangement patterns to mature Vγ2^+^ γδ T cells but that these rearrangements are largely abortive Vγ2-Jγ1 locus rearrangements. This raises an interesting possibility that many lung-resident ILC2s are developmental relics of cells that failed to properly rearrange their TCR genes during neonatal γδ T cell development. This intriguing phenomenon aside, the TCR rearrangements may provide a convenient ontological “fingerprint” that allows tracking of lineage relationships between nILC2s, IL-17-producing ILC2s, and lung ILC3s (76, 77). In short, these gene rearrangement patterns could be used as a genetic barcode to identify whether ILCs switch lineages. With this in mind, it would be of interest to evaluate whether ILC2s and ex-ILC2s (in response to influenza infection, chronic obstructive pulmonary disease (COPD) triggers, or other inflammatory perturbations) display the same genomic rearrangements. Future scRNA-seq analysis during inflammation may reveal the presence of an ILC1-ILC2 subset, ILC2-ILC3 subset, or occult intermediate subsets with mixed transcriptional phenotypes that may be revealed only with appropriate genomic or transcriptional analyses at the single-cell level. Future studies using computational models that better capture continuous variation in ILC transcriptional profiles and explicitly model dependencies among biological topics may help identify key relationships across heterogeneous samples. Finally, the transition potential of ILCs needs to be validated *in vivo* using fate-mapped mice and *in vitro* polarization experiments. While the role of “ex-ILC2s” and “IL-17 producing ILC2s” remains an active area of research, it is also important to investigate whether these populations provide a redundant or physiologically relevant source of IFNγ or IL-17 in inflammatory conditions such as viral infection, helminth infection and asthma.

## Human ILC2s: A jack of all trades involved in immune tolerance and airway diseases

In humans, the three major groups of Lineage^-^CD127^+^ ILCs are conventionally defined based on differential expression of c-Kit (CD117) and CRTH2 (CD294): ILC1s are c-Kit^-^CRTH2^-^, ILC2s are CRTH2^+^c-Kit^+/-^, and ILC3s/ILCps are c-Kit^+^CRTH2^-^ (78, 79). However, the nature of these ILCs is still somewhat controversial and challenging to elucidate because commonly used surface markers (e.g. CRTH2 for ILC2s) may not capture all ILCs (80), and they can exhibit different functions and phenotypes depending on their tissue localization and activation state (81). For example, NKp44^-^ ILC3s are the stereotypical players involved in IL-17 production, but recent reports indicate that human ILC2s are poised to become IL-17 producing cells in response to epithelium-derived cytokines that skew polarization of ILC subsets in the context of different pathologies.

A recent study by Golebski et al. examined the role of ILC2 plasticity in the pathology of chronic rhinosinusitis with nasal polyps (CRSwNP) in cystic fibrosis patients (66). In 2012, Mjosberg et al. previously demonstrated that CRTH2^+^ ILC2s predominate in CRSwNP (16). However, IL-5-producing ILC2s are almost absent in nasal polyps from cystic fibrosis patients (CFwNP) (66). Instead, there is an enrichment of IL-17 producing NKp44^-^ ILC3s in CFwNP. The changes in the accumulation of different ILC subtypes and cytokine profiles between CRSwNP and CFwNP were proposed to be mediated by the transdifferentiation of ILC2s into IL-17 producing ILC3s. The researchers isolated nasal epithelium and designed an air-liquid interface model in an attempt to test this hypothesis. When blood-derived ILC2s were added alongside *S. aureus* and *P. aeruginosa* (common opportunistic bacteria in patients with CF), they stopped producing IL-5 but instead produced significant amounts of IL-17. They found that Th17-polarizing cytokines (IL-1β, IL-23, and TGF-β), generated from the nasal epithelium, stimulated ILC2s to transdifferentiate into IL-17-producing cells. The establishment of a Th17-biased local tissue environment increased RORγt expression in IL-17 producing ILC2 clones. This plasticity was reversible because the addition of IL-4 was sufficient to recover the ILC2-like phenotype and inhibit IL-17 production due to a downregulation in the receptors (*IL1RL1* and *TGFBR1*) necessary for the induction of plasticity. In support of these findings, flow cytometry analysis revealed that blood-derived ILC2s produced IL-17 in response to stimulation with IL-1β, IL-23, and TGF-β (82). The functional plasticity of IL-17 producing ILC2s depended on the downregulation of *GATA3* and the induction of *RORC* given that RORγt blockade diminished IL-17 production *via* ILC2s. Of note, scRNA-seq and flow cytometry analysis revealed the presence of peripheral RORγt^+^c-Kit^+^ ILC2s that expressed CCR6, a marker also found on mouse LTi-like ILC3s, but expressed conventional ILC2-defining genes, including *RORA*, *IL17RB*, and *BCL11B* (82). However, c-Kit^+^ ILC2s were unique because they contained RORγt^+^CCR6^+^ ILC2s and were able to produce IL-17 in response to IL-1β and IL-23 without the need for TGF-β. Conversely, c-Kit^-^ ILC2s were somewhat refractory to the conversion into IL-17-producing ILC3-like cells but could be pushed towards the production of IL-17 with the additional presence of TGF-β. Therefore, the graded expression of c-Kit seems to correlate with the degree of ILC2 plasticity or their mature state.

Another potential mechanism is the recruitment of different ILC subsets from the blood to inflamed tissues. Chen et al. demonstrated that 24 hours after challenge with grass-pollen allergen, ILC2 numbers increased in the sputum but decreased in the blood, suggesting that ILC2s are recruited from the blood upon allergen challenge in asthmatic patients (83). Sputum ILC2s produced significantly more IL-13 post-allergen-challenge. On the other hand, in the periphery, there was no change in IL-13 producing ILC2s, which strongly suggests that ILC2s are recruited from the blood during allergic exacerbation, but the activation of ILC2s occurs in the lung tissue and not the periphery (83). This study illustrates one example of the phenotypic and functional differences between blood and tissue ILCs. Indeed, when ILC2s were isolated from nasal polyps and blood and cultured with IL-2, a cytokine that supports the survival and growth of these cells, only ILC2s derived from the nasal polyps could secrete IL-5 and IL-13, suggesting that tissue-resident ILCs display a more activated or functionally mature state (84). Blood ILC2s display a relatively naive phenotype, and the majority of ILCs in the blood are ILC precursors that only upon migration to tissue may become more activated and differentiate towards a particular ILC subtype (79, 85). Therefore, plasticity may not be the sole mechanism by which ILCs contribute to disease exacerbation, and the recruitment of ILCs remains a possible source of the observed switches in effector ILC programs in human pathophysiology.

The modulation of ILC2 plasticity in humans and in immunotherapy will be beneficial only if plasticity is the causal cellular and molecular pathway by which ILCs regulate disease pathogenesis. While there is not much data on this yet, a recent report demonstrated that a combination of IL-33 and retinoic acid is able to induce IL-10 production by human GATA3^+^ ILC2s, which upregulate Treg-associated genes, including *CTLA4* and *IL2RA* (CD25) (86). This makes these ILC2s resemble cells with a more regulatory phenotype that can potently inhibit both non-IL-10 producing ILC2s as well as Th2 cells. These IL-10-producing ILC2s (so-called regulatory ILCs or ILCregs) were detected in IL-10 reporter mice upon induction of house dust mite (HDM)-mediated lung allergy but were not present at steady state. Golebski et al. further demonstrated that in patients treated with grass pollen immunotherapy for 12 months, peripheral blood ILC2s have greater IL-10 production capacity (compared to blood ILC2s from placebo-treated patients), which indicates that this could be a way for the immune system to dampen the immune response (87).

## ILC3 plasticity in the gut: Controversial players within a continuous spectrum in health and disease

The dominant cellular source of IL-22 in the intestine of humans and mice is RORγt^+^ ILC3s (8, 12, 15, 58). ILC3s are functionally and phenotypically heterogeneous in nature and include a natural cytotoxicity receptor (NCR)^+^ ILC3 subset that can co-express T-bet and RORγt as well as a subset that expresses high levels of CCR6 (originally defined as lymphoid tissue inducer cells that persist postnatally) termed LTi-like ILC3s. NCR^+^ ILC3s are largely absent from intestinal cryptopatches and are, instead, localized primarily to the lamina propria, where they are tuned and poised for IL-22 production (10, 57). Conversely, LTi-like ILC3s are enriched in adult lymphoid tissues (53, 88).

Interestingly, despite a shared core ILC3 program (*Rorc, Il23r*, and *Il22* expression), NCR^+^ ILC3s and LTi-like ILC3s are characterized by subset-specific transcriptional signatures. In fact, global gene expression analysis demonstrates that NCR^+^ ILC3s exhibit a transcriptional profile more like that of ILC1s (*Ifng, Il12rb, Xcl1*, and *Tbx21*) than LTi-like ILC3s, which are T-bet *(Tbx21*) deficient (89). Despite the high level of *Ifng* expression, intestinal NCR^+^ ILC3s produce minimal IFN*γ* at a steady state in WT mice or after *ex vivo* restimulation with IL-12, IL-18 and PMA/ionomycin with brefeldin A (55, 60, 90), but NCR^+^T-bet^+^ ILC3s appear poised for IFN*γ* production as part of the innate defense against infection (91). However, this type 1 functionality may also promote tissue inflammation reminiscent of pathogenic RORγt^+^T-bet^+^ Th17 cells that participate in autoimmune diseases and are dependent on IL-23 (92).

Initial fate-mapping studies in mice have demonstrated that a proportion of adoptively transferred RORγt-fate mapped (FM) intestinal NCR^+^RORγt^+^ ILC3s (isolated from RORγt-GFP reporter mice) can, over time, downregulate RORγt and further upregulate T-bet, thereby becoming so-called “ex-ILC3s” or “ILC1-like” cells, that express NK cell surface markers and transcriptional features associated with type 1 immunity (NK1.1, NKp46, NKG2D, IL-12Rβ2) (53, 54). These ex-ILC3s require IL-15 for their maintenance (32), and are capable of producing IFNγ under distinct inflammatory cytokine conditions (IL-12 and IL-15), implicating them as a major pathological source of IFNγ production. Accordingly, they exacerbate chronic intestinal inflammation in mouse models of CD40-triggered colitis or induce colitis-like pathologies in response to infection (53, 54). These results, only revealed through RORγt-Cre fate mapping strategies, highlight the plasticity of intestinal RORγt^+^ ILC3 subsets and their ability to switch from “homeostatic” ILC3s to IFNγ-producing “inflammatory” ex-ILC3s/ILC1s. While the transition of NCR^+^ ILC3s to ex-ILC3s/ILC1s can be pathogenic, this plasticity is dichotomous and may also be beneficial by evoking protective immunity to certain intracellular pathogens through the production of pro-inflammatory cytokines, namely IFNγ that enables the production of mucus-forming glycoproteins to protect the epithelial barrier (53, 93).

The overexpression of T-bet and progressive loss of RORγt causes a subset of NCR^+^T-bet^+^ ILC3s to transition into an ILC1-like/ex-ILC3 phenotype and display a surface marker phenotype that is akin to cNK cells (Lineage^-^NK1.1^+^NKp46^+^ population), thus making it difficult to distinguish ex-ILC3s with a type 1 effector profile from *bona fide* ILC1s or NK cells (53, 56). Environmental cues such as IL-7R signalling or activation by the microbiota support RORγt expression within NCR^+^ ILC3s and prevent the emergence of ex-ILC3s as seen by the reduced *Tbx21* and *Il22* expression in microbiota-perturbed mice (10, 36, 53, 54, 90). Early studies lacked high-resolution transcriptomic and epigenetic analyses, which are needed to reveal intermediate states and the sum of regulatory elements that dictate each subset’s identity and function.

**Figure 3:**
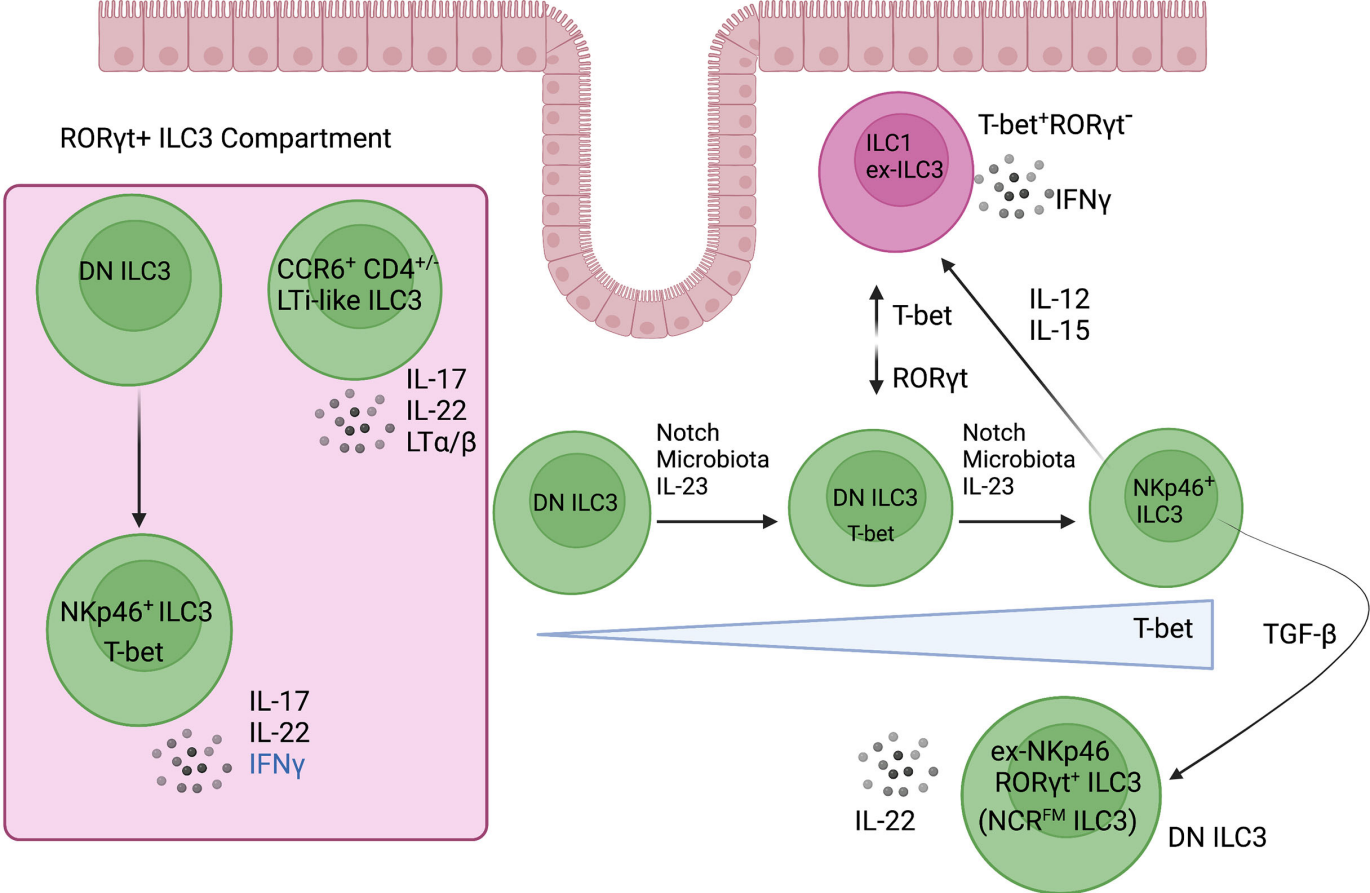
ILC3 plasticity and heterogeneity in the intestine. RORγt^+^ ILC3s are enriched in the intestine where they are heterogeneous and consist of two major subsets. CCR6^+^ ILC3s resemble the fetal lymphoid tissue inducer cells that are required for lymph node organogenesis. The CCR6^-^ ILC3 compartment is distinguished mainly by its expression of the type 1 transcription factor, T-bet. DN (NKp46^-^CCR6^-^) ILC3-precursor cells give rise to NCR^+^ ILC3s, which develop along a T-bet gradient that is controlled by Notch signalling, microbiota, and IL-23. Of note, NCR^+^ ILC3s are poised to express IFNγ under distinct inflammatory cytokine conditions such as IL-12 and IL-15. Created with Biorender.org.

Adding to the diversity within the ILC3 compartment in the intestine, CCR6^-^ ILC3s contain a subset of “double negative” (DN) precursor cells (CCR6^-^NKp46^-^) that upregulate T-bet and give rise to NCR^+^ ILC3s (NKp46^+^RORγt^+^T-bet^+^) (53, 54, 56). Thus, NCR^+^ ILC3s develop along a T-bet gradient (**Figure 3**). In addition, Notch signalling, microbial cues and IL-23 exposure instruct the upregulation of T-bet, thereby regulating the development of NCR^+^ ILC3s and revealing the importance of environmental cues and the cytokine milieu in regulating the fate of ILC3 subsets (53, 56). However, the differential gene signatures defining these intestinal precursor-like DN ILC3s remain challenging, since DN ILC3s can exist in a transitional T-bet^+^ state (between DN ILC3s and NCR^+^ ILC3s) but NKp46^−^ CCR6^−^RORγt^+^ DN ILC3s are still present in *Tbx21* ^−/−^ and germ-free mice, indicating that T-bet is dispensable for their development (53, 60). Therefore, there is a need to identify additional surface markers that distinguish DN ILC3s. Future unbiased scRNA-seq of RORγt-expressing cells using RORγt-reporter mice is required to fully define the role(s) and identity of DN ILC3s and their functional plasticity relative to other RORγt^+^ ILC subsets.

Between the NCR^+^ ILC3 and LTi-like subsets, there lies a range of transcriptional states that has not been previously appreciated. The first scRNA-seq analyses under homeostatic conditions revealed five unique transcriptional states among ILC3s (ILC3a-e) in the small intestinal lamina propria in mice that blurs the boundaries of the current ILC3 subset classification and the diversity within each subset (36). These transcriptional states indicate the possibility of dynamic functional plasticity in response to polarizing signals from the local environment or may represent discrete diversity (36, 40). This could explain the ability of the IL-23 responsive CCR6^+^NCR^-^CD4^-^ LTi-like ILC3 population to co-produce IL-17 and IFNγ in response to *Helicobacter hepaticus* infection in 129SvEv-*Rag* ^-/-^mice (94). However, in the C57BL/6*-Rag^-/-^* strain, this functional plasticity is not induced, and no intestinal inflammation is observed (95). Given that CCR6 and NKp46 are used as mutually exclusive markers to discriminate LTi-like ILC3s and NCR^+^ILC3s, it is unclear whether IL-17 producing LTi-like ILC3s do indeed have functional plasticity to produce IFNγ, seemingly in contrast to reports indicating that NCR^+^T-bet^+^ ILC3s represent the ILC3 subset with the capacity to produce IFNγ upon transition to ex-ILC3/ILC1-like cells under distinct inflammatory conditions (40, 53, 54).

NCR^+^ ILC3s have an unconventional transcription factor profile that consists of master regulators, RORγt, T-bet, and GATA3 (53, 56, 96). Of note, although NKp46 is not stably expressed in ILC3s, fate-mapping studies using *R26* ^eYFP^ *Ncr1-*iCre revealed a population of NCR^-^ ILC3s (FM^+^) that still expressed YFP, indicating that NKp46 expression has occurred in their life history (97). Using mice that could report and ablate IL-22 expression in NCR^+^ ILCs, NCR^-^ ILC3s (FM^+^) were shown to produce IL-22. In line with this finding, conditional gene targeting in NKp46^+^ ILCs using *Ncr1* ^creGFP^ mice demonstrated that fate-mapped NKp46^FM^ ILC3s (ex-NKp46^+^ ILC3s) in the small intestinal lamina propria produced more IL-22 than NCR^+^ ILC3s at steady-state and expressed higher levels of c-Kit (CD117), reminiscent of DN ILC3s (98). Importantly, these ex-NKp46^+^ ILC3s did not produce IL-17A or IFNγ, indicating that these murine ‘ex-NKp46 ILC3s’ revert to a DN ILC3 phenotype and are distinct from ex-ILC3s/ILC1s.

Although T-bet is important for the development of NCR^+^ ILC3s, NCR^-^ ILC3s (FM^+^) are still present in a T-bet deficient background (97). Therefore, T-bet is not required for the transition of NCR^-^ ILC3s to NCR^+^ ILC3s but may be required for the maintenance of NCR^+^ ILC3s. In addition to T-bet, Notch signalling also plays an important role in the balance of NCR^-^ ILC3s and NCR^+^ ILC3s (97, 99). When pure NCR^+^ ILC3s are sorted and cultured in the presence of Dll1 (Notch ligand), the majority of NCR^+^ ILC3s retain NKp46 expression. In the absence of Notch, a substantial fraction of NCR^+^ ILC3s lose NKp46 expression, which is associated with a downregulation of T-bet (97). Notch2 signals found in the tissue microenvironment are critically important in the transition of DN ILC3s into NCR^+^ ILC3s by inducing the expression of T-bet and RORγt (99). Indeed, bone marrow reconstitution experiments and mouse models in which the Notch pathway was abrogated or constitutively activated revealed a direct, cell-intrinsic action whereby DN ILC3 precursor cells differentiate into NCR^+^ ILC3s (**Figure 3**) (99). Of note, TGF-β plays an important role in regulating the balance between NCR^-^ ILC3s and NCR^+^ ILC3s because it antagonizes Notch signalling and suppresses the transition of DN ILC3s to NCR^+^ ILC3s. This highlights the ability of ILC3 subsets to modulate their transcription factor profiles, effector functions, and phenotype in response to environmental signals (97).

## What we know about the transcriptional regulatory circuits governing ILC3 functional phenotype and plasticity

ILC3s are critically dependent on the master transcription factor RORγt for their development, but the transient inhibition of RORγt expression in mature ILC3s does not affect core ILC3 functions, such as IL-22 production, likely because there are other key-ILC associated transcription factors regulating ILC function and phenotype (100). A recent study utilizing 5X polychromic reporter mice (*Id2* ^BFP^ *Gata3* ^hCD2^ *Rora* ^Teal^ *Bcl11b* ^tdTom^ *Rorc* ^Kat^) demonstrated that all ILC subsets express RORα, including small intestinal ILC1s/ex-ILC3s and this finding concurs with other gene expression analyses (89, 101). NK cells, however, failed to express RORα (101). Crucially, our previous findings revealed that RORα plays a key role in preserving ILC3 characteristics (102, 103). We demonstrated that dysregulated RORα expression reshapes the transcriptional spectrum of ILCs and attenuates expression of core ILC3-signature genes, including downregulation of *Rorc*, *Il23r*, and *Il1r* - an aberrant gene signature likely reflecting dysfunctional ILC3s that are unable to detect an inflammatory milieu. Moreover, in a *Salmonella*-driven model of Crohn’s disease-like fibrosis, we showed that RORα-deficient mice were protected: RORα inactivation dampened Th17/ILC3-type cytokine production, including IL-17 and IL-22. The role of RORα in preserving ILC3 phenotype and function indicates that ILC fate and/or plasticity is dictated by an intricate balance and maintenance of transcription factor programs.

**Figure 4:**
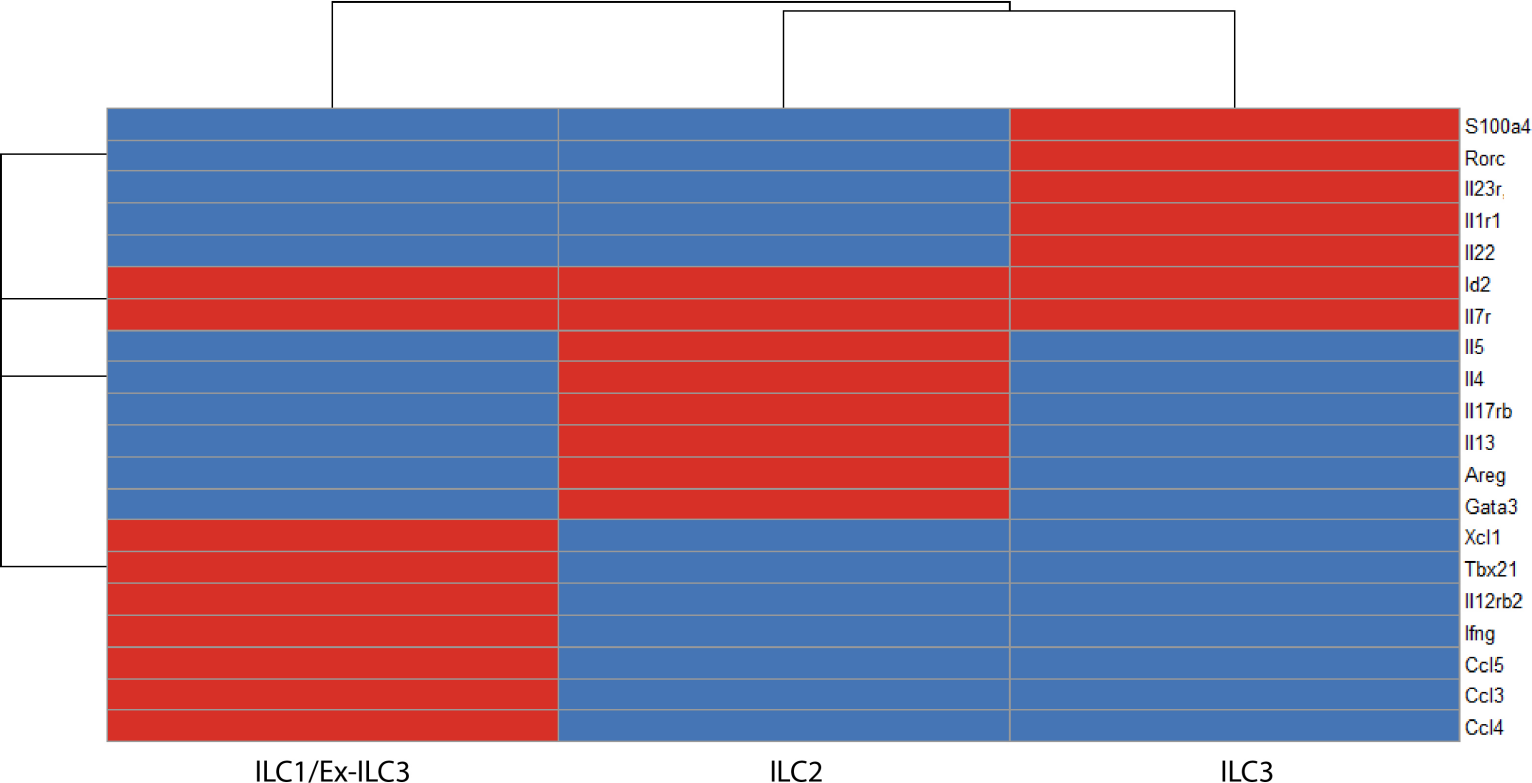
A binary matrix of ILC signature genes in the intestinal lamina propria (red = expressed, blue = not expressed). Common genes that distinguish ILC subsets in the intestinal lamina propria based on ScRNA-seq reports (36, 60, 103, 104). *Id2* and *Il7r* are core ILC genes. ILC1s/ex-ILC3s express *Tbx21*, *Ifng*, *Il12rb2*, *Ccl5*, *Gzmc.* ILC2s express high levels of *Gata3*, *Il17rb*, *Areg*, *Il5*, and *Il13*. ILC3s express *Rorc*, *Il1r1*, and *Il23r* (ILC3 program), which makes these cells exquisitely sensitive to stimulation with IL-23 and thus produce high levels of IL-22 on a per cell basis.

Another recent study investigated the roles of key-ILC associated transcription factors in regulating ILC3 heterogeneity, function, and phenotype using scRNA-seq of ILCs isolated from the small intestinal lamina propria from four inducible transgenic mouse models that allow combinatorial deletion of RORγt, RORα and T-bet in ILCs (60). Five ILC “superclusters” were identified from all genotypes: ILC1s/Ex-ILC3s, ILC2s, NCR^+^ ILC3s, LTi-like ILC3s, and an “unknown” ILC cluster. Of note, RORγt depletion together with RORα led to the complete loss of NCR^+^ ILC3s and a concomitant expansion of ex-ILC3s/ILC1s, which was associated with enhanced T-bet expression and a downregulated ILC3 program. Although the deletion of RORγt was associated with a loss in ILC3-related genes such as *Rorc, Il1r*, and *Il23r*, the full acquisition of a T-bet orchestrated type 1 immunity program and trans-differentiation towards an ex-ILC3/ILC1-like population required the deletion of RORα. These ex-ILC3s converge upon the same transcriptional and functional state as ILC1s (**Figure 4**).

Among ILC3s, c-Maf expression is highly correlated with that of T-bet, and conditional deletion of *Maf* along with fate mapping RORγt^+^ ILC3s demonstrated that c-Maf regulates the balance of lineage-defining transcription factors, RORγt and T-bet (55). Specifically, c-Maf is downregulated with ILC3 to ILC1 conversion and deletion of c-Maf results in the upregulation of T-bet and concomitant downregulation of RORγt. *Rorc* fate-mapping approaches demonstrated that ex-ILC3s are increased in the *Maf* knockout, indicating increased conversion in the absence of c-Maf. Therefore, it appears that c-Maf functions to restrain this ILC3 to ILC1 cell plasticity, acting as a gatekeeper for the acquisition of type 1 features in ILC3s (55).

These regulatory circuits and co-expressed transcription factors appear to play a critical role in the identity and plasticity of ILC3s and possibly other ILC subsets and offer an exciting area for future research and manipulation.

## The spectrum of ILC3-ILC1-like cells in humans

It is noteworthy that the ILC3 to ILC1 transitions identified in mouse models have also been observed in humans. At steady-state conditions, ILC3s represent the most abundant ILC subset in the human intestine, whereas the frequency of ILC1s is extremely low. When highly purified NKp44^+^ ILC3s from fetal gut or tonsils are cultured with IL-2 and IL-12 in the presence of feeder cells, ILC3s stop producing IL-22 and lose expression of NKp44 and c-Kit (ILC3 phenotypic markers), but instead start producing large amounts of IFNγ, suggesting the differentiation of NKp44^+^ ILC3s towards CD127^+^ ILC1s (61, 62). This shift has been proposed as a contributor to the observed changes between non-inflamed tissue versus inflamed regions of patients with Crohn’s disease where there is a substantial increase in IFNγ-producing CD127^+^ ILC1s at the cost of homeostatic IL-22 producing NKp44^+^ ILC3s in the inflamed intestine (61).

Beyond the conventional IL-22 producing CD103^-^NKp44^+^ ILC3s (ILC3a) and IFNγ-producing CD103^+^NKp44^+^ ILC1s (ILC1a), two additional ILC subsets are detected in inflamed tonsils. These CD103^+^NKp44^+^ ILCs included CD196^+^CD300LF^+^ and CD300LF^−^CD196^+^ subsets referred to as ILC3b and ILC1b, respectively (105). Functional analysis of clones derived from these four ILC3-ILC1 subsets revealed a gradient in which the capacity to produce IFNγ increased from ILC3a to ILC3b to ILC1b, to ILC1a clones, which were exclusively IFNγ-producing cells. These four ILC3-ILC1 subsets were subjected to scRNA-seq and RNA velocity analysis to interrogate the whole spectrum of ILC3-ILC1-like cells and predict the future state of ILC3-ILC1 subsets in the human tonsils and lamina propria of ileal specimens (105). In this report, an intermediate cluster expressing *IL7R*, *CD300LF*, and *KLRD1* (CD94) manifested itself along the ILC3-ILC1 trajectory and appeared in a vector heading towards an IFNγ-producing ILC1 cluster that expressed *TBX21* and *IFNG*, indicative of plasticity. However, this work does not exclude the possibility that certain ILC subsets may directly derive from the differentiation of undetected rare progenitor cells. While there is strong evidence for ILC3s becoming more plastic and perhaps more ILC1-like cells in inflammatory bowel disease (61, 62), there could be other pathways regulating this shift in ILC composition, as discussed. Additionally, there certainly could be oxidative stress and cell death occurring in the context of ILC3s in inflammatory bowel disease (IBD) patients, which to the best of our knowledge, has not been carefully examined and warrants further investigation.

## NK cells and ILC1s in the liver: A brief insight on the presence of locally maintained progenitors versus plasticity

NK cells and ILC1s are both defined as Lineage^-^NK1.1^+^NKp46^+^ IFNγ-producing cells that are driven by the transcription factor T-bet (106). Despite these similarities, the mouse liver represents one location in which one can clearly discriminate NK cells from ILC1s based on differential expression of CD49a and CD49b (107, 108). ILC1s are CD49a^+^CD49b^-^ whereas NK cells are CD49a^-^ CD49b^+^. Liver ILC1s are further defined by the expression of CD200R1, TRAIL, and CD69, which are all not found on NK cells. On the other hand, NK cells express the transcription factor EOMES, which distinguishes them from ILC1s. Surprisingly, in contrast to NK cells, liver ILC1s are not reconstituted by bone marrow cells (108). Instead, fetal liver cells are more efficient in reconstituting the liver ILC1 compartment. Intriguingly, fetal liver and adult liver contains a population of Lineage^-^Sca-1^+^Mac-1^+^ cells with preferential ILC1 progenitor, over NK cell progenitor, activity. It is noteworthy that ablation of the capacity of NK cells and ILC1s to produce IFNγ attenuates the number of liver ILC1s, suggesting that IFNγ is a prerequisite for liver ILC1 development. A previous study using *Ncr1^cre^Eomes^floxed^* mice showed that ablation of EOMES in NKp46^+^ cells depleted NK cells but had no impact on liver ILC1s (109). In aggregate, these results suggest that, like myeloid cells, which are derived in part from progenitors from embryonic life, a vanguard of fetal Lineage^-^Sca-1^+^Mac-1^+^ ILC1 precursors from the fetal liver seed this tissue and persist during adulthood. Thus, liver ILC1s develop locally *via* an IFNγ− dependent loop (108).

This, in turn, has led to speculation that NK cells can convert into ILC1-like cells (110, 111). For example, Cortez et al. demonstrated that ablation of SMAD4 in NKp46-expressing cells can induce NK cells to acquire an ILC1-like gene signature, including *Itga1* (CD49a) and *Tnfsf10* (TRAIL) expression (111). These ex-NK cells (ILC1-like) upregulate CD49a and were unable to control NK-cell-dependent containment of B16 lung metastasis. The presence of locally maintained ILC1 progenitors in the liver raises the question of whether the observed shift towards an ILC1-like phenotype by TGF-β imprinted SMAD4-deficient NK cells is in part due to a pathway that affects these progenitors, instead of plasticity.

## Methodologies to ascertain ILC plasticity

ILC immune responses feature complex heterogeneity and transitions among cell states within what would be considered a single cell type. Therefore, to truly evaluate plasticity, there must be corroborative evidence that consists of techniques such as single-cell transcriptomic studies accompanied by lineage tracing, barcoding and epigenetic analyses.

For example, a recent study by Bielecki et al. conducted scRNA-seq of ILCs (CD45^+^Lineage^-^CD90^+^) sorted from naïve and psoriatic skin of WT and *Rag1^-/-^* mice and identified a dense continuum of functional states and graded gene expression with overlapping expression of type 2 and type 3 genes (47). Due to the continuous variability in scRNA-seq data, this study used a probabilistic topic modelling to infer biological “topics”, including “repressive/quiescent”, “type2/ILC2-like”, and “mixed type 3/pro-inflammatory ILC3-like”, to describe the transcriptional profile in each cell. This revealed a spectrum of ILC states that was not previously anticipated. In fact, this spectrum shifted to a type 2/3 hybrid phenotype upon disease induction, hinting that classical IL-5 and IL-13 producing ILC2s transition to a new, pathology-associated mixed ILC2/ILC3-like subset. These mixed states and ILC2-ILC3 plasticity were experimentally validated *in vivo* by IL-5 fate-mapping and IL-22^BFP^/IL-17A^GFP^ reporter mice that demonstrated a proportion of cells that expressed type 3 cytokines also previously expressed IL-5 (47). This suggests that some of the ILC3-like cells that arise after disease induction express the ILC2 program at an earlier point in their lifetime. Single-cell assay for transposase-accessible chromatin sequencing (scATAC-seq) of ILC populations from untreated skin showed open chromatin at transcription start sites for *Il5* and *Il13* alongside open chromatin at regulatory elements for *Il22*, *Il17a*, *Il17f*, and *Il13*, indicating that steady-state skin ILCs are epigenetically poised to become ILC3-like cells.

## Discussion

Inflammatory diseases elicit a markedly different tissue environment compared to that of healthy, non-inflamed tissues, and these conditions can be remarkably disease-specific. At baseline, ILC subsets are present in characteristic frequencies in distinct anatomical locations, and they exhibit tissue-specific phenotypes and effector programs (81, 112). However, multiple studies suggest that different ILC subsets are expanded in the inflammatory environment and that ILCs can dramatically change their phenotype and function from that observed at homeostasis (39, 113). These perturbation-induced changes in mature effector ILC populations play a crucial role in the pathogenesis of upper and lower airway diseases as well as gut and skin inflammatory diseases. Thus, a critical question is how these shifts in frequency and phenotype occur mechanistically: *de novo* generation of cells *in situ* from precursors? Influx from a novel population from peripheral sites? Plastic reprogramming of local ILC populations? Ultimately, these mechanisms are key to understanding how inflammatory and repair functions are initiated, executed, and resolved and how they can be targeted to ameliorate inflammation or facilitate tissue repair. It is important to note that a combination of mechanisms might contribute to shifts in effector ILC populations. For instance, even if mature effector cells exist in a much more fluid state than as discrete entities, immature precursors may also directly contribute to the ILC compartment without the need for cell-state transitions.

**Figure 5:**
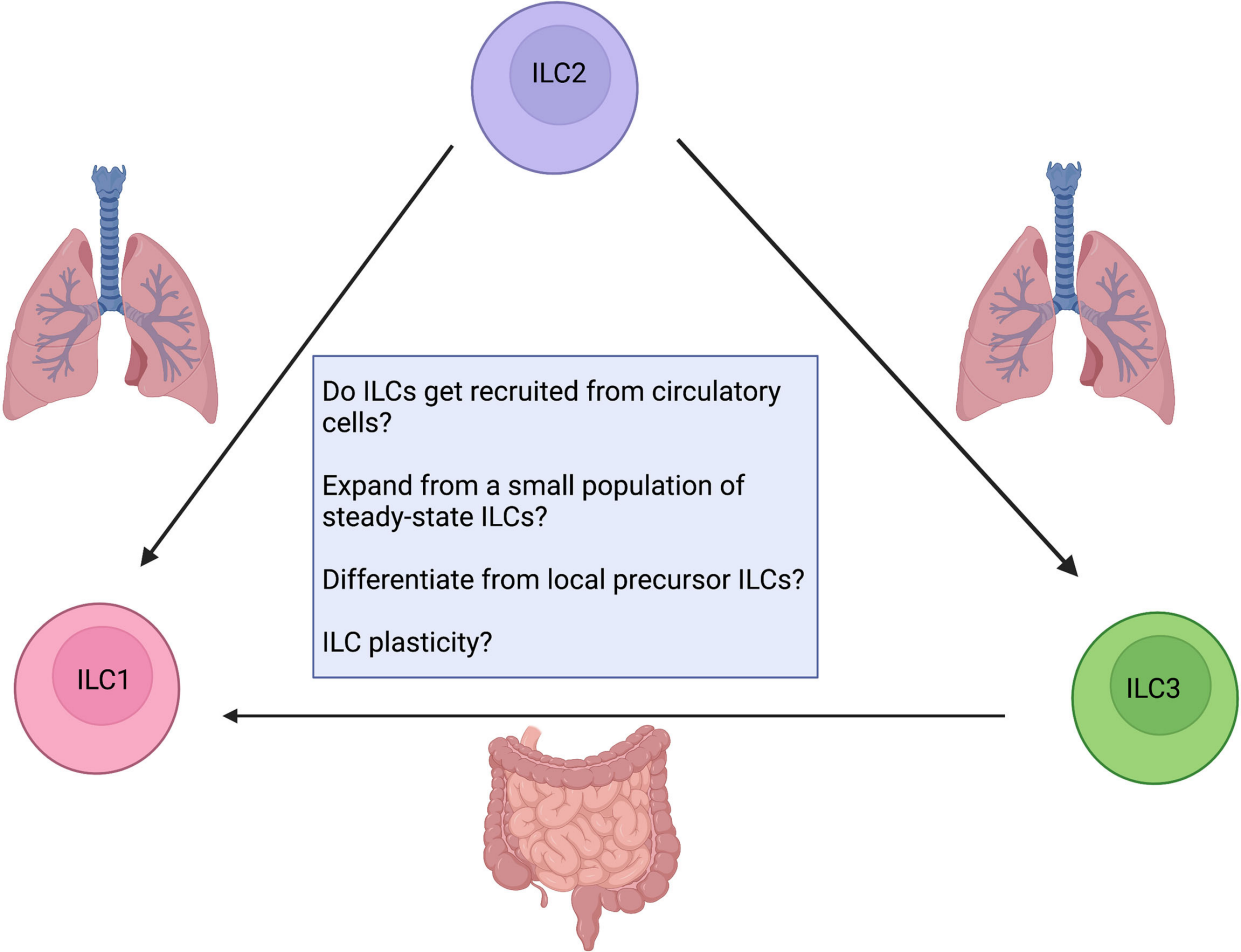
Confounding factors when addressing ILC plasticity. How do ILCs contribute to inflammation? Are they recruited from circulatory cells? Do they expand from a small undetected steady-state population or locally maintained ILC precursors? Are tissue-resident ILCs plastic? Future work aimed at developing critical animal models as reporter assays for fate mapping and lineage tracing will enable us to answer these questions. Created with Biorender.org.

The skewed ILC composition during chronic imbalances and inflammatory diseases in humans and mice has been proposed to be caused by “ILC plasticity”. The concept of ILC plasticity suggests that the identity of ILC subsets is not set in stone and that, in response to potent local microenvironmental stimuli, these cells can transdifferentiate to produce different cytokines and adopt alternate cell-fates. However, as noted above, there are several confounders in many of these studies (**Figure 5**). We do not yet know to what extent the accumulation of different ILC subsets reflects *in situ* proliferation of tissue-resident ILCs, including expansion from rare undetected steady-state populations, versus recruitment of ILCs from the peripheral blood. Alternatively, these shifts in ILC subset numbers could result from the differentiation of locally-maintained precursor-like cells upon receiving appropriate localizing signals in tissue. Of note, each of those mechanisms has been shown to be relevant in different tissues or disease contexts. For example, the lung contains a range of *Il18r1* ^+^ ILC progenitors (43), and when perturbed by *N. brasilinesis* infection, these precursors are pushed to transition from *Il18r1* ^high^ *Tcf7* ^high^ to *Gata3* ^high^ *Il1rl1* ^high^ through a proliferating transit-amplifying stage to give rise to a plethora of effector ILC2s (44). In terms of recruitment from circulatory cells, IL-25-activated ILC2s can upregulate S1P receptors and acquire the ability to enter the lymphatic vessels of villi (48). Once they enter the periphery, BATF-dependent iILC2 cells can accumulate in systemic sites of infection such as the lungs to contribute to tissue repair and orchestrate the re-establishment of barrier integrity (48, 49). Campbell et al. further found that *Trichinella spiralis*, an entirely gastrointestinal-dwelling mucosal parasite that never enters the lungs, caused intestinal iILC2s to proliferate and these effector IL-13-producing ILC2s moved into the blood to provide systemic innate protection in the lungs (51). Thus, there is evidence for dramatic local ILC differentiation and rapid influx from peripheral sites as a confounder to many studies of “plasticity”.

Given the competing (or complementary) mechanistic explanations of current ILC plasticity studies, we advocate for a more critical approach that examines each case before inferring ILC plasticity and that, ideally, *bona fide* plasticity is confirmed through single-cell assays and cell tracking. Future studies are needed to further clarify lineage relationships, transition potential, and whether ILC plasticity can be manipulated for improved treatment of clinical disease. It is important to conduct appropriate epigenetic and transcriptional analysis at the single-cell level, along with functional assays and fate-mapping strategies to confidently claim “plasticity”. While ILC plasticity may allow for flexible immunity to intracellular pathogens (beneficial), it has also been associated with autoimmunity (pathogenic), particularly inflammatory bowel disease in humans (61, 62, 113). Therefore, improved experimental rigour and the use of genomic rearrangements may offer additional insight into the extent of each subset’s adaptability in response to the local tissue microenvironment and previous findings need to be revisited to avoid misleading interpretations.

## Author contributions

AK contributed to conceptualization, topic curation, and wrote the review, and designed the figures and table. KM contributed to conceptualization, topic curation, writing, editing, and approved the manuscript. SS read, edited, and approved the manuscript. MH contributed to the elaboration of the figures and read, edited, and approved the manuscript. All authors contributed to the article and approved the submitted version.

## Funding

This work was funded by grant numbers PJT-148681 and PJT-156235 from the Canadian Institutes of Health Research (CIHR). SS was supported by an AllerGen Network Centre of Excellence and CIHR Frederick Banting & Charles Best Canada Graduate Scholarship–Master’s Program (CGS-M) Scholarship.

## Conflict of interest

The authors declare that the research was conducted in the absence of any commercial or financial relationships that could be construed as a potential conflict of interest.

## Publisher’s note

All claims expressed in this article are solely those of the authors and do not necessarily represent those of their affiliated organizations, or those of the publisher, the editors and the reviewers. Any product that may be evaluated in this article, or claim that may be made by its manufacturer, is not guaranteed or endorsed by the publisher.
